# Relationship between parental marital conflict and malevolent creativity propensity among junior school students: roles of deviant peer affiliation and self-control

**DOI:** 10.3389/fpsyt.2026.1794219

**Published:** 2026-05-13

**Authors:** Mingzhe Zhao, Yan Liu

**Affiliations:** 1 School of Education, Huazhong University of Science & Technology, Wuhan, China; 2 Department of Humanities and Management, Hebei University of Chinese Medicine, Shijiazhuang, China

**Keywords:** deviant peer affiliation, early adolescence, interparental conflict, malevolent creativity propensity, self-control

## Abstract

This study explored the relationship between parental marital conflict and malevolent creativity propensity among junior school students, as well as the roles of deviant peer affiliation and self-control on that relationship. A sample of 947 junior school students was surveyed using the Children’s Perception of Marital Conflict Scale, Deviant Peers Affiliation Scale, Brief Self Control Scale, and Malevolent Creativity Behavior Scale. The results indicated that parental marital conflict was positively associated with students’ malevolent creativity propensity, and deviant peer affiliation partially mediated the relationship between parental marital conflict and malevolent creativity propensity. In addition, self-control moderated the second stage of the mediation of deviant peer affiliation in the link between parental marital conflict and malevolent creativity propensity. Ecological systems theory, social learning theory, and Self-Determination theory were used to explain our findings, while limitations and future research directions were discussed in detail.

## 1 Introduction

Although the general public typically views creativity as a positive and desirable process (1), the motivation behind the creative process has long been overlooked. Based on the moral nature of the underlying motivation, researchers have categorized creativity into benevolent creativity and malevolent creativity (2). Malevolent creativity is typically defined as the creative ideas which are generated to purposefully inflict material, mental, or physical harm on others (3, 4). Although common malevolent ideas may lead to detrimental results, malevolent ideas that are also creative may pose an even greater threat. This increased danger may be related to the fact that creative malevolent ideas are unusual, thus making them more difficult for individuals to detect, prevent, and respond to (5). For this reason, in recent years, researchers have primarily explored malevolent creativity from several perspectives, including individual personality traits (e.g., aggression, conscientiousness, the dark triad; 5–7), emotional factors (e.g., anger; 8) and situational factors (e.g., social threat, bullying victimization, categorizing as an out-group member, and facing infectious disease threat; 9–12). These findings suggest that malevolent creativity occurs especially in individuals with a propensity for aggressiveness and among those facing harmful behaviors and intentions from others.

Since adolescence is a critical period for the development of individual creativity (13), providing proper guidance to students during this stage may be associated with the moral orientation of their creative motivation, both in the present and in the future. As so far, few studies have examined factors associated with adolescents’ malevolent creativity propensity (11). Based on ecological systems theory, the family and the peer groups are two important subsystems that are associated with adolescent development (14). These subsystems are interconnected rather than developing independently, and risk factors in one subsystem can increase adolescents’ exposure to risk factors in the other, thereby contributing to the emergence of problematic behaviors. However, the effect of microsystems varies depending on the individual traits (14). Therefore, this study explored the relationship between parental marital conflict and malevolent creativity propensity as well as the possible inner mechanism (deviant peer affiliation) and protective factor (self-control).

### 1.1 The relationship between parental marital conflict and malevolent creativity propensity

Ecological systems theory posits that the family is an important microsystem in children’s development (14). The quality of parents’ marital relationship is closely associated with the development of adolescents (15). Parental marital conflict refers to verbal arguments or physical aggression between spouses caused by disagreements or other factors (16). When parents experience conflict, they may redirect their negative emotions toward their children through aggression and blame, which can trigger adolescents’ negative emotions. For example, when children are exposed to a family environment characterized by parental conflict over an extended period, it can, to some extent, positively predict their depression (15) and anxiety (17, 18). These negative emotions can increase children’s aggressive tendencies (19). Meanwhile, such emotions may, through cognitive persistence—engaging in a deeper and more systematic exploration of a conceptual category—activate remote concepts, thereby generating malevolent and creative ideas (20). In addition, according to social learning theory, individuals who have been exposed to parental marital conflict over time may imitate their parents’ aggressive behaviors because they may perceive this behavioral pattern as reasonable and acceptable (21). Researchers have proposed that individuals with low levels of moral reasoning have stronger moral self-regulatory ability to ensure consistency between actual behavior and moral standards (22), whereas those with high moral disengagement believe that harming others in novel ways is morally permissible, which is associated with higher levels of malevolent creativity (23). Therefore, we hypothesize that parental marital conflict is positively associated with malevolent creativity propensity among junior school students (H1).

### 1.2 The mediating role of deviant peer affiliation

According to ecological systems theory (14), disruptive factors in the family microsystem can spill over into other systems. Peers constitute another important microsystem beyond the family. Deviant peer affiliation refers to adolescents’ selective affiliation with peers who exhibit serious problem behaviors (e.g., cheating, substance abuse, and aggressive behavior; 24–26). According to Self-Determination Theory (27), if parents fail to meet their children’s basic psychological needs (e.g., self-esteem and love), it may increase the likelihood that children will seek psychological compensation through other means. Previous studies have found that parental marital conflict positively predicts deviant peer affiliation among junior school students (28, 29). In addition, individuals who affiliate with deviant peers tend to maintain cognitive and behavioral consistency with their peers, resulting in higher moral disengagement and more aggressive behavior (30). Previous research has found that deviant peer affiliation predicts adolescents’ bullying behavior by increasing moral disengagement (31), and both moral disengagement and aggression were related to malevolent creativity (5, 23). Therefore, we hypothesize that deviant peer affiliation mediates the relationship between parental marital conflict and malevolent creativity propensity (H2).

### 1.3 The moderating role of self-control

Although parental marital conflict and deviant peer affiliation may both be associated with malevolent creativity propensity, these associations may vary across individuals. As Bronfenbrenner (14) proposed, the power of proximal processes varies depending on the individual traits. This study proposes that the effect of parental marital conflict on malevolent creativity propensity—mediated via deviant peer affiliation—may differ depending on the adolescents’ level of self-control. Self-control refers to the ability to inhibit or alter one’s thoughts, emotions, and motivations to pursue long-term goals (32). According to the strength model of self-control (33), short-term self-control activities temporarily deplete self-control resources, which leads to the state of ego depletion, and thus may lead to more problematic behaviors. Previous research suggests that individuals with high trait self-control can indirectly reduce emotional and cognitive stress by inhibiting automatic cognitive processing and promoting reflective coping strategies (34). When facing parental conflict, adolescents with high self-control may reduce adolescents’ negative emotions and hostile cognitions by cognitively inhibiting automatic reactions and engaging in reflective reappraisal; in contrast, individuals with low self-control have limited self-control resources (33) and may find it difficult to eliminate the negative emotions and cognition responses induced by parental conflict, thereby increasing hostility toward others and, through cognitive persistence, activating creative processes and exhibiting higher levels of malevolent creativity propensity. Therefore, we hypothesize that self-control moderates the relationship between parental marital conflict and malevolent creativity propensity (H3).

Adolescents who are exposed to prolonged parental conflict may experience a state of depleted self-control resources because they may attempt to regulate their emotional arousal and then maintain family harmony by alleviating tensions between their parents during conflicts (35). The general theory of crime posits that low self-control is a fundamental trait common to people engaging in deviant peer affiliation (36). An individual with low self-control lacks the ability to regulate emotions and impulsive behaviors (37), which may lead them to be alienated from conventional peers and increase the likelihood of affiliating with deviant peers. Empirical studies strongly support the association between low self-control and high levels of deviant peer affiliation (38). Therefore, low self-control trait adolescents may have a higher level of deviant peer affiliation because their self-control resources may be depleted after exposure to parental conflict. We hypothesize that self-control moderates the relationship between parental marital conflict and deviant peer affiliation (H4).

Furthermore, adolescents with high self-control may be less likely to mimic the behaviors of their deviant peers because they have more resources to regulate their cognition and behavior that are in line with social standards (33). Individuals with high self-control may inhibit the impulse to pursue immediate social rewards and instead focus on long-term goals, thereby achieving volitional disengagement when facing peer temptation (39). Empirical results show that, for adolescents with low or moderate trait self-control, peer deviance significantly predicts increases in antisocial behavior; however, for adolescents with high trait self-control, peers’ negative influence is no longer statistically significant (39). Another study has also found that self-control can moderate the effect of peers’ deviant behavior on adolescents’ aggressive behavior (29). Therefore, we hypothesize that self-control moderates the relationship between deviant peer affiliation and malevolent creativity propensity (H5).

## 2 The present study

**Figure 1:**
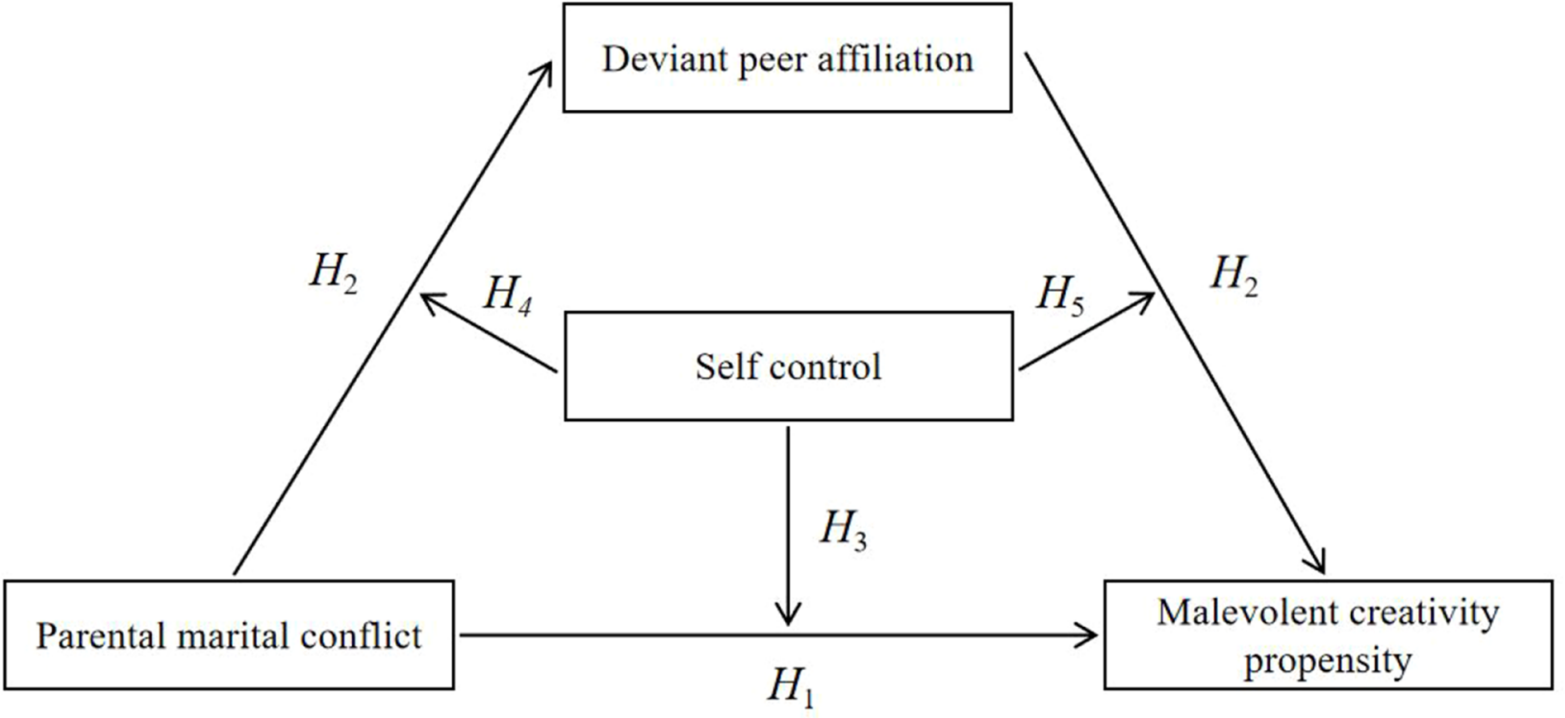
Moderated mediation model.

Taken together, although previous studies suggest that malevolent creativity occurs especially in people with a propensity for aggressiveness and among those facing behaviors and intentions from others that may have harmful consequences, few researchers have focused on adolescents (11), a group in the developmental stage in which creativity is highly malleable (13). Based on the ecological systems theory and the strength model of self-control, this study proposes a moderated mediation model (**Figure 1**) to examine the relationship between parental marital conflict and malevolent creativity propensity among junior school students, as well as the roles of deviant peer affiliation and self-control on that relationship.

The following hypotheses were formulated:

H1 Parental marital conflict is positively associated with malevolent creativity propensity among junior school students.

H2 Deviant peer affiliation mediates the relationship between parental marital conflict and malevolent creativity propensity.

H3 Self-control moderates the relationship between parental marital conflict and malevolent creativity propensity.

H4 Self-control moderates the relationship between parental marital conflict and deviant peer affiliation.

H5 Self-control moderates the relationship between deviant peer affiliation and malevolent creativity propensity.

## 3 Methods

### 3.1 Participants and procedure

Convenience sampling was used to select a junior middle school in central China. The schools we surveyed were located in urban areas and admitted adolescent students from both urban and rural regions. Cluster convenience sampling was adopted to choose all students in the first- and second-grades classes. The school’s mental health teachers administered the questionnaires during school hours with the consent of the students’ guardians. The questionnaires were distributed by class and completed via an online platform in the school computer lab. After reading the study description and data confidentiality principles, students were informed that they could refuse to participate or withdraw at any time. Those who chose to participate completed the questionnaire after reading the informed consent form and received small gifts as compensation.

Finally, 947 students completed the survey. Among those students, 494 were boys (52.17%) and 453 were girls (47.84%), 497 were from urban areas (52.48%) and 450 were from rural areas (47.52%), with a mean age of 12.23 ± 0.84 years. Besides, based on the statistics in our study, 229 adolescents (24.18%) perceived obvious parental marital conflict (self-reported scores exceeding the median on a 4-point Likert scale). This suggests that some students in our study are experiencing relatively obvious parental marital conflict. This study was reviewed and approved by the Institute’s Ethics Committee.

### 3.2 Measures

#### 3.2.1 Children’s perception of marital conflict scale

The Children’s Perception of Marital Conflict Scale was developed to assess parental marital conflict (40). In this study, the Chinese version of Children’s Perception of Marital Conflict Scale was adopted (16). This scale consists of 17 items, such as “When Mom and Dad argue, they get really angry”. Respondents rated their agreement on a 4-point Likert scale ranging from 1 (totally disagree) to 4 (totally agree). All the items were averaged as the total scores of the Chinese version of Children’s Perception of Marital Conflict Scale (range from 1 to 4), with higher scores indicating elevated levels. In the current study, the Cronbach’s α for this scale was 0.91. The confirmatory factor analysis showed a good structural validity: χ²= 591.43, *df* = 119, CFI = 0.94, TLI = 0.92, RMSEA = 0.07, SRMR = 0.04.

#### 3.2.2 Deviant peers affiliation scale

Adolescent affiliation with deviant peers was assessed with eight items adapted from prior published questionnaires (25, 41). Deviant peer behaviors included smoking, alcohol use, cheating on school tests, stealing or shoplifting, misbehaving, Internet addiction, skipping or cutting school, and physical and verbal aggression. Respondents indicated how many of their friends had shown each of the eight deviant behaviors (e.g., “How many of your friends got drunk?”) on a 5-point scale ranging from 1 (none) to 5 (almost all). Responses were averaged across the eight items (range from 1 to 5), with higher scores representing greater deviant peer affiliation. In the current study, the Cronbach’s α for this scale was 0.78. The confirmatory factor analysis showed a good structural validity: χ²= 56.40, *df* = 20, CFI = 0.99, TLI = 0.98, RMSEA = 0.04, SRMR = 0.01.

#### 3.2.3 Brief self-control scale

The Brief Self Control Scale was developed to assess self-control (42). In this study, the Chinese Brief Self Control Scale was adopted to assess self-control (43). This scale consists of 7 items and two subscales: self-discipline and impulse control, such as “I am good at resisting temptation” and “I do certain things that are bad for me, if they are fun”. Respondents rated their agreement on a 5-point Likert scale ranging from 1 (not at all) to 5 (very much). All the items were averaged as the total scores of the Brief Self Control Scale (range from 1 to 5), with higher scores indicating greater levels. In the current study, the Cronbach’s α for this scale was 0.76. The confirmatory factor analysis showed a good structural validity: χ²= 57.20, *df* = 13, CFI = 0.97, TLI = 0.94, RMSEA = 0.06, SRMR = 0.05.

#### 3.2.4 Malevolent creativity behavior scale

The Malevolent Creativity Behavior Scale (MCBS) was used to assess individual malevolent creativity propensity (44). It contains 13 items and consists of three subscales: hurting people, lying, and playing tricks, such as “How often do you think about ideas to take revenge when treated unfairly?”, “How often do you fabricate lies to simplify a problem situation?”, and “How often do you have ideas about how to pull pranks on others?”. Respondents rated their agreement on a 5-point Likert scale ranging from 0 (never) to 4 (usually). All the items were averaged as the total scores of the Malevolent Creativity Behavior Scale (range from 0 to 4), with higher scores indicating greater levels. In the current study, the Cronbach’s α for this scale was 0.91. The confirmatory factor analysis showed a good structural validity: χ²= 257.51, *df* = 62, CFI = 0.97, TLI = 0.96, RMSEA = 0.06, SRMR = 0.03.

### 3.3 Statistical analysis

We used SPSS 25.0 to output descriptive statistics and Pearson correlation coefficients. Subsequently, we used Hayes’ (45) PROCESS macro in SPSS to test the mediation and moderated mediation models described below, with 5,000 bootstrap resamples. All variables were z-standardized prior to the PROCESS analyses. This tool is specifically designed for conducting path analysis of mediation and moderation effects involving manifest variables. When the 95% bootstrap confidence interval from the bootstrapping analysis does not include zero, the indirect effect is considered significant.

## 4 Results

### 4.1 Common method bias test

Given that all variables in this study were obtained from self-report measures, the results may be susceptible to common method bias. To assess common method bias, we employed Harman’s single-factor test (46). The analysis revealed eight factors with eigenvalues greater than 1, with the first factor accounting for 21.95% of the total variance, which is below the critical threshold of 40%. This result suggests that common method bias is unlikely to be a significant issue in this study.

### 4.2 Descriptive statistics

As summarized in **Table 1**, parental marital conflict demonstrated statistically significant positive correlations with both deviant peer affiliation and malevolent creativity propensity, alongside a negative correlation with self-control. Furthermore, deviant peer affiliation displayed positive associations with malevolent creativity propensity and negative associations with self-control. Notably, self-control exhibited a negative relationship with malevolent creativity propensity.

**Table 1:** Pearson’s correlations, means, and standard deviations.

Variables	*M*	*SD*	1	2	3	4
1. Parental marital conflict	1.58	0.67	—			
2. Deviant peer affiliation	2.06	0.63	0.25***	—		
3. Self control	3.15	0.75	-0.42***	-0.31***	—	
4. Malevolent creativity propensity	1.46	0.45	0.29***	0.26***	-0.26***	—

*N* = 947, ***p < 0.001.

### 4.3 Relationship between parental marital conflict and malevolent creativity propensity: testing the moderated mediation effect

We first conducted a simple mediation analysis to test whether deviant peer affiliation mediated the association between parental marital conflict and malevolent creativity propensity, initially excluding self-control as a moderator. The results showed that parental marital conflict positively predicted deviant peer affiliation (*a* = 0.25, *SE* = 0.03, *t* = 8.35, *p* < 0.001, 95% CI = [0.19,0.31]). When both parental marital conflict and deviant peer affiliation were included in the regression equation, parental marital conflict (*c’* = 0.19, *SE* = 0.03, *t* = 5.97, *p* < 0.001, 95% CI = [0.13,0.25]) and deviant peer affiliation (*b* = 0.24, *SE* = 0.03, *t* = 7.47, *p* < 0.001, 95% CI = [0.18,0.31]) both positively predicted malevolent creativity propensity. The bias-corrected non-parametric percentile Bootstrap test indicated that deviant peer affiliation significantly mediated the relationship between parental marital conflict and malevolent creativity propensity (*ab* = 0.06, 95% CI = [0.04,0.09]), the mediating effect accounted for 24.64% of the total effect.

We then tested the hypothesized moderated mediation model, in which self-control was specified to moderate the direct effect of parental marital conflict on malevolent creativity propensity and the two component paths of the indirect effect. Regarding moderation (see **Table 2**), the interaction between parental marital conflict and self-control did not significantly predict malevolent creativity propensity, indicating that self-control did not moderate the direct effect. Next, the interaction between parental marital conflict and self-control was not significant in predicting deviant peer affiliation, suggesting that the first stage of the mediation was not moderated. Finally, the interaction between deviant peer affiliation and self-control significantly predicted malevolent creativity propensity, indicating that self-control moderated the second stage of the mediation.

**Table 2:** Moderated mediation analysis of the association between parental marital conflict and malevolent creativity propensity.

Predictors	M: Deviant peer affiliation				Y: Malevolent creativity propensity			
	Estimate	*SE*	*t*	95% CI	Estimate	*SE*	*t*	95% CI
Parental marital conflict	0.20	0.03	6.29***	0.14, 0.26	0.10	0.03	3.19**	0.04, 0.16
Deviant peer affiliation					0.17	0.03	5.40***	0.11, 0.23
Self-control	-0.18	0.03	-5.87***	-0.24, -0.12	-0.34	0.03	-11.31***	-0.40, -0.28
Parental marital conflict×self-control	0.03	0.03	1.21	-0.02, 0.08	-0.03	0.03	-1.29	-0.08, 0.02
Deviant peer affiliation×self-control					-0.07	0.03	-2.68**	-0.12, -0.02
*R*	0.32				0.48			
*R^2^*	0.10				0.23			
*F*	36.32***				56.35***			

Estimate, regression coefficient (in standard deviation units); variables were z-standardized prior to analysis; *SE*, standard error; 95% CI, 95% confidence interval. ***p < 0.001, **p < 0.01.

Because the interaction between parental marital conflict and self-control was nonsignificant in both the mediator and outcome equations, no further probing of this interaction was performed. We then examined whether the indirect association between parental marital conflict and malevolent creativity propensity via deviant peer affiliation varied across levels of self-control by estimating conditional indirect effects at low (−1 *SD*), mean, and high (+1 *SD*) self-control using bootstrapping. The indirect effect was significant at low self-control (conditional indirect effect = 0.04, Boot*SE* = 0.01, Boot 95% CI = [0.02, 0.07]) and at the mean level of self-control (conditional indirect effect = 0.03, Boot*SE* = 0.01, Boot 95% CI = [0.02, 0.06]), but was not significant at high self-control (conditional indirect effect = 0.02, Boot*SE* = 0.02, Boot 95% CI = [−0.01, 0.06]), indicating that higher self-control attenuated the indirect association via deviant peer affiliation.

**Figure 2:**
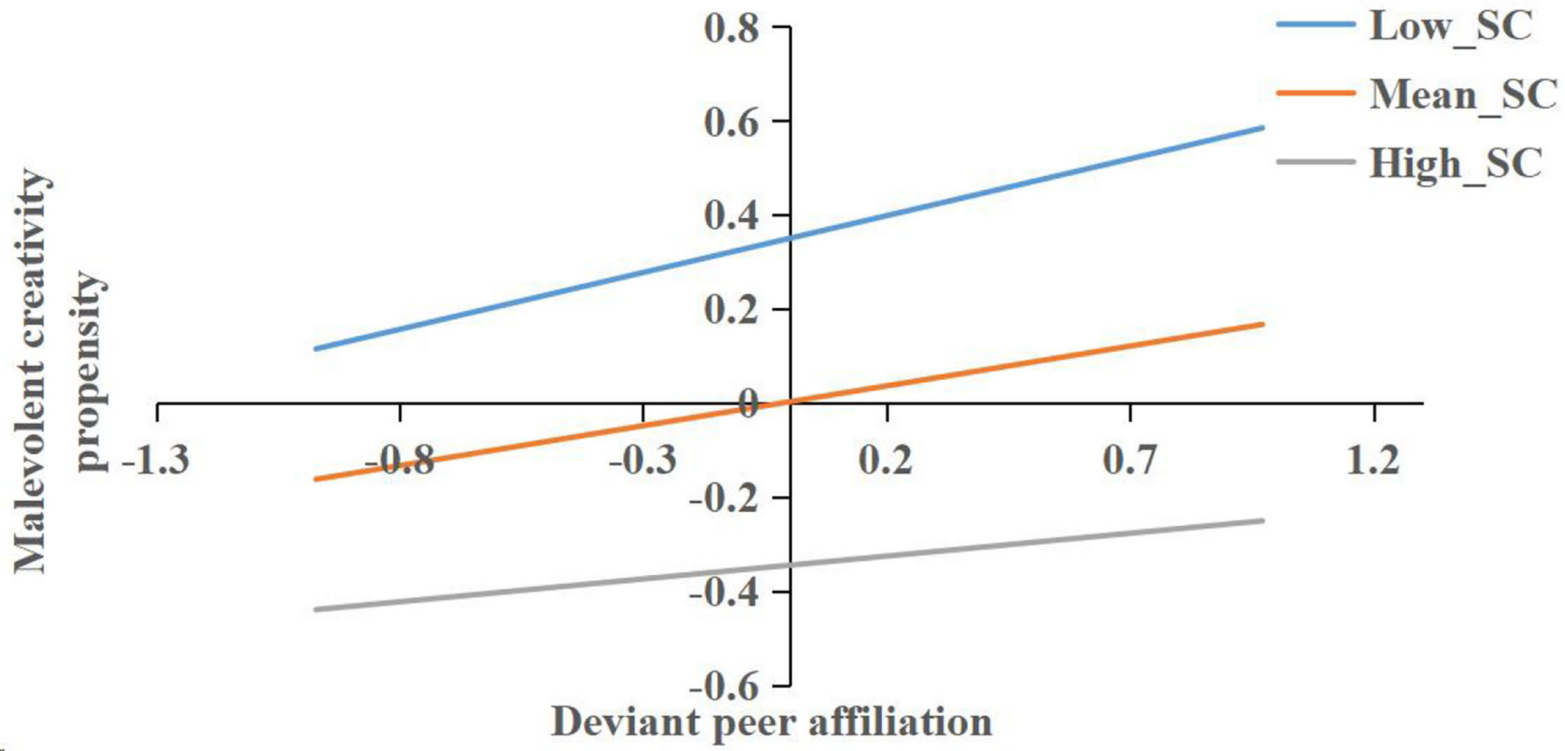
Conditional effect of deviant peer affiliation on malevolent creativity propensity at low, mean, and high levels of self-control. Variables are z-standardized; SC, self-control.

To clarify the significant interaction (deviant peer affiliation × self-control) predicting malevolent creativity propensity, we conducted a simple slope (conditional effect) analysis by estimating the association between deviant peer affiliation and malevolent creativity propensity at low (−1 *SD*), and high (+1 *SD*) levels of self-control (**Figure 2**). Deviant peer affiliation was more strongly associated with malevolent creativity propensity when self-control was low (Estimate = 0.24, *SE* = 0.04, *t* = 6.26, *p* < 0.001, 95% CI = [0.17, 0.32]) than when self-control was at the mean level (Estimate = 0.17, *SE* = 0.03, *t* = 5.40, *p* < 0.001, 95% CI = [0.11, 0.23]) or high (Estimate = 0.10, *SE* = 0.04, *t* = 2.21, *p* = 0.03, 95% CI = [0.01, 0.18]), consistent with a buffering role of self-control on the association between deviant peer affiliation and malevolent creativity propensity.

## 5 Discussion

This study investigated the relationship between parental marital conflict and malevolent creativity propensity among junior school students, focusing on the underlying moderated mediation mechanism. Results indicated that parental marital conflict significantly and positively predicted malevolent creativity propensity. Furthermore, deviant peer affiliation played a partial mediating role in this relationship. Crucially, the latter part of this indirect pathway was moderated by self-control, such that the adverse influence of deviant peers was significantly attenuated for students with high self-control. These results support the hypotheses proposed in this study and align with the perspective of ecological systems theory, which posits that individual development is influenced by multiple nested subsystems such as family and peers.

### 5.1 The relationship between parental marital conflict and malevolent creativity propensity among junior school students

The present study found that parental marital conflict significantly and positively predicted malevolent creativity propensity, thus supporting Hypothesis 1. When parents experience conflict, they may redirect their negative emotions onto their children. The more difficulties a family encounters in fulfilling its functions, or the poorer the effectiveness of family functioning, the more likely individuals are to adopt aggression toward others as the best strategy for dealing with problems (29, 47). In addition, the Dual Pathway to Creativity model suggests that negative affect can elicit a more analytical, bottom-up processing style and promote cognitive persistence (20). Because parental marital conflict has been associated with heightened negative emotions among adolescents (15, 17, 18), such emotional states may facilitate creativity propensity when they generate malevolent ideas. Besides, repeated exposure to parents’ negative affect and maladaptive conflict tactics may socialize aggressive scripts and hostile interpretations (21), which may lead adolescents to not only generate malevolent creative ideas but also implement malevolent creativity behaviors (48). Taken together, parental marital conflict may foster malevolent creativity propensity by simultaneously increasing malevolent motives and facilitating creative ideation processes.

### 5.2 The mediating role of deviant peer affiliation in the relationship between parental marital conflict and malevolent creativity propensity among junior school students

The results indicate that parental marital conflict was indirectly associated with malevolent creativity propensity via deviant peer affiliation, thus supporting Hypothesis 2. On one hand, in family environments filled with parental marital conflict, parents tend to neglect the care and supervision of their children. This leads to a reduction in emotional communication among family members. Excessive exposure to such environments makes it difficult for individuals to build trusting relationships with the outside world and increases the likelihood of deviating from mainstream social circles during adolescence (27). As a result, they may turn to deviant peers to cope with the negative emotions caused by conflict (27). Through sharing experiences of deviant behavior, they seek emotional support and attempt to fill the emotional void left by their families (49). On the other hand, according to group socialization theory, peer factors play a crucial role in individual development and socialization (29, 30). During interactions with deviant peers, individuals may imitate their externalizing problem behaviors, thus shaping similar behaviors in themselves (30). Deviant peers may engage in more aggression (50) as well as criminal behaviors (51), and both are related to malevolent creativity (3, 5, 52). Thus, adolescents facing parental marital conflict may learn and imitate their peers’ malevolent creative ideas. Notably, deviant peer affiliation only partially accounted for the association between parental marital conflict and malevolent creativity propensity. This pattern suggests that although peer processes represent an important pathway, additional mechanisms (e.g., heightened negative affect or hostile cognitions) may also contribute to malevolent creativity propensity (11, 52).

### 5.3 The moderating role of self-control in the relationship between parental marital conflict and malevolent creativity propensity among junior school students

This study found that self-control did not significantly moderate the associations between parental marital conflict and deviant peer affiliation or malevolent creativity propensity. A possible explanation is that the family environment is among the earliest and most influential contexts to which adolescents are exposed (28). Accordingly, perceptions of parental marital conflict may exert a relatively pervasive influence on adolescents’ socioemotional functioning, increasing the likelihood of affiliating with deviant peers and engaging in malevolent ideation across levels of self-control.

The results further revealed that self-control moderates the pathway from deviant peer affiliation to malevolent creativity propensity. Deviant peer affiliation significantly and positively predicted malevolent creativity propensity among adolescents with low self-control, whereas this effect was weaker among those with high self-control, thus supporting Hypothesis 5. Previous studies have found positive associations between deviant peer affiliation and negative emotions (e.g., depression; 53), maladaptive cognitions (e.g., moral disengagement; 54), and problem behaviors (e.g., aggressive behavior; 54). This may be because deviant peer affiliations may be ill-equipped to provide warmth and support for adolescents, and adolescents who affiliate with deviant peers may also tend to use moral disengagement tactics to minimize feelings of guilt and avoid self-sanctions (53, 54). Based on the strength model of self-control (33), adolescents with high self-control have more resources to regulate negative emotions and inhibit maladaptive thoughts. Previous studies also found that self-control can moderate the relationship between deviant peer affiliation and moral disengagement as well as aggression (29, 54). Therefore, adolescents with high self-control may have less malevolent creativity propensity because they have more resources to manage negative emotions and cognitions arising from deviant peer contexts. Taken together, self-control is less likely to alter adolescents’ exposure to parental conflict or deviant peer contexts, but it plays a key role in buffering the translation of deviant peer-related risks into malevolent creativity propensity, consistent with a person-by-context perspective embedded in ecological systems theory (14).

### 5.4 Limitations

Firstly, the use of a convenience sampling approach, with all participants are recruited from a single junior middle school, may result in a lack of diversity on demography (e.g., geographical distribution). Besides, this study didn’t collect data of family socioeconomic status among adolescents. Consequently, the external validity of the study is constrained. To address this limitation, we plan to expand the scope of future research by including students from diverse geographical regions, age groups and family socioeconomic status to further examine whether the relationship between parental marital conflict and malevolent creativity propensity is moderated by these demographic variables; secondly, this is a cross-sectional study. Any causal relationship based on the associations observed in our study should be inferred cautiously. Future research could use experimental or longitudinal designs (e.g., parental marital conflict priming or cross-lagged panel models) to clarify the temporal ordering of the observed associations and to test whether changes in perceived parental marital conflict are followed by changes in deviant peer affiliation and malevolent creativity propensity; thirdly, previous studies found that daily activities (e.g., sports) could increase the level of self-control (55). Future research may explore whether self-control training could influence the level of malevolent creativity propensity among students experiencing high parental marital conflict; finally, all data were collected through self-report questionnaires. Therefore, recall bias and the subject expectancy effect may be difficult to avoid. Besides, the measurement of malevolent creativity was a self-reporting scale but not a traditional tool such as Alternative Uses Task. This scale can only reflect the self-cognitive propensity of students. Behavioral tasks (e.g., malevolent creativity task; Perchtold-Stefan et al., 2021) could be added to make the conclusion more realistic and reliable in the future.

## 6 Conclusion

In summary, this study found that: (1) parental marital conflict among junior school students is significantly positively correlated with deviant peer affiliation and malevolent creativity propensity, and deviant peer affiliation is significantly positively correlated with malevolent creativity propensity; (2) deviant peer affiliation partially mediates the relationship between parental marital conflict and malevolent creativity propensity; (3) self-control moderates the mediating role of deviant peer affiliation in the pathways between parental marital conflict and malevolent creativity propensity. Specifically, deviant peer affiliation was more strongly associated with malevolent creativity propensity among adolescents with low self-control traits, but the above effect was weaker among adolescents with high self-control traits.

## Data Availability

The raw data supporting the conclusions of this article will be made available by the authors, without undue reservation.

## References

[1] Andreasen N Ramchandran K . Creativity in art and science: Are there two cultures? Dialogues Clin Neurosci. (2012) 14:49–54. doi: 10.31887/DCNS.2012.14.1/nandreasen. PMID: 22577304 PMC3341649

[2] Cropley DH Cropley AJ Kaufman JC Runco MA . The dark side of creativity. New York, NY, USA: Cambridge University Press (2010).

[3] Cropley DH Kaufman JC Cropley AJ . Malevolent creativity: A functional model of creativity in terrorism and crime. Creativity Res J. (2008) 20:105–15. doi: 10.1080/10400410802059424. PMID: 37339054

[4] Cropley DH Kaufman JC White AE Chiera BA . Layperson perceptions of malevolent creativity: The good, the bad, and the ambiguous. Psychol Aesthetics Creativity Arts. (2014) 8:400–12. doi: 10.1037/a0037792. PMID: 27371692

[5] Harris DJ Reiter-Palmon R . Fast and furious: The influence of implicit aggression, premeditation, and provoking situations on malevolent creativity. Psychol Aesthetics Creativity Arts. (2015) 9:54–64. doi: 10.1037/a0038499. PMID: 27371692

[6] Jia X Wang Q Lin L . The relationship between childhood neglect and malevolent creativity: The mediating effect of the dark triad personality. Front Psychol. (2020) 11:613695. doi: 10.3389/fpsyg.2020.613695. PMID: 33391134 PMC7772220

[7] Lee SA Dow GT . Malevolent creativity: Does personality influence Malicious divergent thinking? Creativity Res J. (2011) 23:73–82. doi: 10.1080/10400419.2011.571179. PMID: 37339054

[8] Perchtold‐Stefan CM Fink A Rominger C Papousek I . Creative, antagonistic, and angry? Exploring the roots of malevolent creativity with a real‐world idea generation task. J Creative Behav. (2021) 55:710–22. doi: 10.1002/jocb.484. PMID: 34690361 PMC8518065

[9] Baas M Roskes M Koch S Cheng Y De Dreu CK . Why social threat motivates malevolent creativity. Pers Soc Psychol Bull. (2019) 45:1590–602. doi: 10.1177/0146167219838551. PMID: 30931827

[10] Du X Zhao Y Zhang K . The influence of group categorization and common ingroup identity on malevolent creativity, benevolent creativity, and neutral creativity. Thinking Skills Creativity. (2024) 54:101686. doi: 10.1016/j.tsc.2024.101686. PMID: 38826717

[11] Tong D Shi Y Gu X Lu P . Bullying victimization and Malevolent Creativity in Rural adolescents: the longitudinal Mediational role of hostile attribution. Cyberpsychol Behav Soc Networking. (2024) 27:420–5. doi: 10.1089/cyber.2023.0499. PMID: 38511278

[12] Zhao M Zhang K Du X . The effect of infectious disease threat on malevolent creativity. J Intell. (2022) 10:111. doi: 10.3390/jintelligence10040111. PMID: 36412791 PMC9680440

[13] Barbot B . Perspectives on creativity development: new directions for child and adolescent development Vol. 151. Hoboken, NJ, USA: John Wiley & Sons (2016). 10.1002/cad.20146 26994720

[14] Bronfenbrenner U . The Ecology of Human Development: Experiments by Nature and Design. Cambridge, MA, USA: Harvard University Press (1979).

[15] Fan H Zhu Z Miao L Liu S Zhang L . Impact of parents' Marital conflict on adolescent depressive symptoms: A moderated mediation model. psychol Dev Educ. (2018) 34:481–8. doi: 10.16187/j.cnki.issn1001-4918.2018.04.12

[16] Chi L Xin Z . The revision of children's perception of marital conflict scale. Chin Ment Health J. (2003) 17:554–6.

[17] Adare AA Zhang Y Hu Y Wang Z . Relationship between parental marital conflict and social anxiety symptoms of Chinese college students: mediation effect of attachment. Front Psychol. (2021) 12:640770. doi: 10.3389/fpsyg.2021.640770. PMID: 34552521 PMC8450334

[18] Li T Dong H . Children's perception of interparental conflict and anxiety: the mediating effect of attention to positive and negative information and the moderating effect of gender. psychol Dev Educ. (2023) 39:488–96. doi: 10.16187/j.cnki.issn1001-4918.2023.04.04

[19] Zhang C Zhang Q Zhuang H Xu W . The reciprocal relationship between depression, social anxiety and aggression in Chinese adolescents: The moderating effects of family functioning. J Affect Disord. (2023) 329:379–84. doi: 10.1016/j.jad.2023.02.134. PMID: 36870452

[20] Nijstad BA De Dreu CK Rietzschel EF Baas M . The dual pathway to creativity model: Creative ideation as a function of flexibility and persistence. Eur Rev Soc Psychol. (2010) 21:34–77. doi: 10.1080/10463281003765323. PMID: 37339054

[21] Bandura A Walters RH . Social learning theory Vol. 1. . Englewood Cliffs, NJ: Prentice Hall (1977) p. 141–54.

[22] Bandura A . Social cognitive theory of self-regulation. Organizational Behav Hum Decision Processes. (1991) 50:248–87. doi: 10.1016/0749-5978(91)90022-L

[23] Shi Z Zhou Z Tian L Zhu Y Liu C Xu L . What causes malevolent creative people to engage in malevolent behaviors? Mediating role of moral disengagement and moderating effects of conscience. Thinking Skills Creativity. (2023) 49:101329. doi: 10.1016/j.tsc.2023.101329. PMID: 38826717

[24] Fergusson DM Wanner B Vitaro F Horwood LJ Swain-Campbell N . Deviant peer affiliations and depression: Confounding or causation? J Abnormal Child Psychol. (2003) 31:605–18. doi: 10.1023/A:1026258106540. PMID: 14658741

[25] Fergusson DM Horwood LJ . Prospective childhood predictors of deviant peer affiliations in adolescence. J Child Psychol Psychiatry. (1999) 40:581–92. doi: 10.1111/1469-7610.00475. PMID: 10357164

[26] Keijsers L Branje S Hawk ST Schwartz SJ Frijns T Koot HM . Forbidden friends as forbidden fruit: Parental supervision of friendships, contact with deviant peers, and adolescent delinquency. Child Dev. (2012) 83:651–66. doi: 10.1111/j.1467-8624.2011.01701.x. PMID: 22181711

[27] Deci EL Ryan RM . Self-determination theory. Handb Theories Soc Psychol. (2012) 1:416–36. doi: 10.1007/978-94-007-0753-5_2630

[28] Liu S Fan H Wang Z Song M Teng H . Parental marital conflict and bulling among middle school students: roles of deviant peer affiliation and sensation seeking. psychol Dev Educ. (2025) 41:235–44. doi: 10.16187/j.cnki.issn1001-4918.2025.02.09

[29] Su P Zhang W Yu C Liu S Xu Y Zhen S . Influence of parental marital conflict on adolescent aggressive behavior via deviant peer affiliation: A moderated mediation model. psychol Dev Educ. (2017) 40:1392–8. doi: 10.16719/j.cnki.1671-6981.20170618

[30] Harris JR . Where is the child's environment? A group socialization theory of development. psychol Rev. (1995) 102:458–89. doi: 10.1037/0033-295X.102.3.458. PMID: 27371692

[31] Wang X Yang J Wang P Zhang Y Li B Xie X . Deviant peer affiliation and bullying perpetration in adolescents: The mediating role of moral disengagement and the moderating role of moral identity. J Psychol. (2020) 154:199–213. doi: 10.1080/00223980.2019.1696733. PMID: 31815608

[32] Mei SL Chai JX Guo JH . Subjective well-being and internet addiction of adolescents: Mediating roles of self-esteem and self-control. psychol Dev Educ. (2015) 31:603–9. doi: 10.16187/j.cnki.issn1001-4918.2015.05.12

[33] Baumeister RF Vohs KD Tice DM . The strength model of self-control. Curr Dir psychol Sci. (2007) 16:351–5. doi: 10.1111/j.1467-8721.2007.00534.x. PMID: 40046247

[34] Galla BM Wood JJ . Trait self‐control predicts adolescents’ exposure and reactivity to daily stressful events. J Pers. (2015) 83:69–83. doi: 10.1111/jopy.12083. PMID: 24354437

[35] Wang M Sun S Liu X Yang Y Liu C Huang A . Interparental conflict and early adolescent depressive symptoms: parent-child triangulation as the mediator and grandparent support as the moderator. J Youth Adolescence. (2024) 53:186–99. doi: 10.1007/s10964-023-01923-2. PMID: 38091163 PMC10761398

[36] Gottfredson MR Hirschi T . A general theory of crime. Stanford, CA, USA: Stanford University Press (1990).

[37] Cho S Hong JS Sterzing PR Woo Y . Parental attachment and bullying in South Korean adolescents: Mediating effects of low self-control, deviant peer associations, and delinquency. Crime Delinquency. (2017) 63:1168–88. doi: 10.1177/0011128717714968

[38] Liu F Chui H Chung MC . The effect of parent–adolescent relationship quality on deviant peer affiliation: The mediating role of self-control and friendship quality. J Soc Pers Relat. (2020) 37:2714–36. doi: 10.1177/0265407520937358

[39] Gardner TW Dishion TJ Connell AM . Adolescent self-regulation as resilience: Resistance to antisocial behavior within the deviant peer context. J Abnormal Child Psychol. (2008) 36:273–84. doi: 10.1007/s10802-007-9176-6. PMID: 17899361

[40] Grych JH Seid M Fincham FD . Assessing marital conflict from the child's perspective: The Children's Perception of Interparental Conflict Scale. Child Dev. (1992) 63:558–72. doi: 10.1111/j.1467-8624.1992.tb01646.x. PMID: 1600822

[41] Li D Li X Wang Y Zhao L Bao Z Wen F . School connectedness and problematic Internet use in adolescents: A moderated mediation model of deviant peer affiliation and self-control. J Abnormal Child Psychol. (2013) 41:1231–42. doi: 10.1007/s10802-013-9761-9. PMID: 23695186

[42] Morean ME DeMartini KS Leeman RF Pearlson GD Anticevic A Krishnan-Sarin S . Psychometrically improved, abbreviated versions of three classic measures of impulsivity and self-control. psychol Assess. (2014) 26:1003. doi: 10.1037/pas0000003. PMID: 24885848 PMC4152397

[43] Luo T Cheng L Qin L Xiao S . Reliability and validity of chinese version of brief self-control scale. Chin J Clin Psychol. (2021) 29:83–6. doi: 10.16128/j.cnki.1005-3611.2021.01.017

[44] Hao N Tang M Yang J Wang Q Runco MA . A new tool to measure malevolent creativity: The malevolent creativity behavior scale. Front Psychol. (2016) 7:682. doi: 10.3389/fpsyg.2016.00682. PMID: 27242596 PMC4870273

[45] Hayes AF . Introduction to mediation, moderation, and conditional process analysis: A regression-based approach. New York, NY, USA: Guilford Publications (2017).

[46] Zhou H Long L . Statistical remedies for common method biases. Adv psychol Sci. (2004) 12:942–50.

[47] Lindsey EW Chambers JC Frabutt JM Mackinnon‐Lewis C . Marital conflict and adolescents' peer aggression: The mediating and moderating role of mother‐child emotional reciprocity. Family Relations. (2009) 58:593–606. doi: 10.1111/j.1741-3729.2009.00577.x. PMID: 40046247

[48] Xu X Zhao J Xia M Pang W . I can, but I won't: Authentic people generate more malevolently creative ideas, but are less likely to implement them in daily life. Pers Individ Dif. (2021) 170:110431. doi: 10.1016/j.paid.2020.110431. PMID: 38826717

[49] Li M Gan X Jin X . Marital conflicts and internet gaming disorder in adolescents: multiple mediations of deviant peer affiliation and neuroticism. Chin J Clin Psychol. (2020) 28:354–8. doi: 10.16128/j.cnki.1005-3611.2020.02.028

[50] Ellis WE Zarbatany L . Peer group status as a moderator of group influence on children’s deviant, aggressive, and prosocial behavior. Child Dev. (2007) 78:1240–54. doi: 10.1111/j.1467-8624.2007.01063.x. PMID: 17650136

[51] Fergusson DM Swain-Campbell NR Horwood LJ . Deviant peer affiliations, crime and substance use: A fixed effects regression analysis. J Abnormal Child Psychol. (2002) 30:419–30. doi: 10.1023/A:1015774125952. PMID: 12108769

[52] Cheng R Lu K Hao N . The effect of anger on malevolent creativity and strategies for its emotion regulation. Acta Psychologica Sin. (2021) 53:847–60. doi: 10.3724/SP.J.1041.2021.00847

[53] Yu C Liao X Ni X Wang H . How and when deviant peer affiliation influence non-suicidal self-injury? Testing a longitudinal moderated serial mediation model among Chinese early adolescents. Child Psychiatry Hum Dev. (2025), 1–10. doi: 10.1007/s10578-025-01825-3. PMID: 40156667

[54] Wang X Wang S Zeng X . Does deviant peer affiliation accelerate adolescents' cyberbullying perpetration? Roles of moral disengagement and self‐control. Psychol Schools. (2023) 60:5025–40. doi: 10.1002/pits.23037. PMID: 41531421

[55] Shachar K Ronen-Rosenbaum T Rosenbaum M Orkibi H Hamama L . Reducing child aggression through sports intervention: The role of self-control skills and emotions. Children Youth Serv Rev. (2016) 71:241–9. doi: 10.1016/j.childyouth.2016.11.012. PMID: 38826717

